# P-1945. Genomic Surveillance of SARS-CoV-2 Variants among Children Enrolled in the New Vaccine Surveillance Network (NVSN) - Kansas City

**DOI:** 10.1093/ofid/ofae631.2104

**Published:** 2025-01-29

**Authors:** Dithi Banerjee, Stephanie Gummersheimer, Anjana Sasidharan, Benjamin R Clopper, Heidi L Moline, Rangaraj Selvarangan

**Affiliations:** Children's Mercy Hospital, Overland Park, Kansas; Children's Mercy Hospital, Overland Park, Kansas; Childrens Mercy Hospital, Missouri, Kansas; US Centers for Disease Control & Prevention, Buffalo, New York; Centers for Disease Control and Prevention, Atlanta, Georgia; Children’s Mercy Kansas City, Kansas City, Missouri

## Abstract

**Background:**

SARS-CoV-2 has evolved since its emergence in late December 2019, with evidence for increased contagiousness, evasion of immunity and missed detection by diagnostic assays. SARS-CoV-2 genomic surveillance in children is important to evaluate impact of the variants on pediatric COVID-19 disease.

**Methods:**

During April 2021-October 2023, the New Vaccine Surveillance Network conducted active, prospective surveillance for acute respiratory illness (ARI) among children (< 18 years). Children with ARI symptoms were enrolled and had mid-turbinate swab samples tested for SARS-CoV-2, either by clinical standard of care or by surveillance testing. SARS-CoV-2 positive samples with cycle threshold values < 30 were processed using PacBio HiFiViral SARS-CoV-2 kit and sequenced on a Sequel IIe system. Consensus FASTA sequences were generated using SMRT^®^ Link (V13.0); clades and lineages were assigned using Nextclade (https://clades.nextstrain.org/). Additionally, number of mapped reads, median coverage, list of S gene mutations, and multiple strains probability were determined.

**Results:**

We attempted sequencing of 204 samples and sequenced 150 (73.5%) samples; 130/150 samples had >95% coverage. Sequencing failed in 52 (25.5%) samples (< 70% coverage) and 2 (0.9%) without any genome coverage. SMRT link analysis flagged 29 samples (14.2%) as having multiple strains (probability >0.95). Median genome coverage was 99.5% (IQR: 98–99.6], with a median of 12768 reads/ sample (IQR: 2913 - 50502). Twenty-eight Delta strains from 2 clades and 7 lineages (mostly clade 21J - 27, 18%) and 122 Omicron strains from 13 clades and 24 lineages (mostly clade 21K - 61, 40.7%) were identified. All identified strains had >1 S gene substitution, while 149 (99.3%) and 66 (44%) had S gene deletions and insertions, respectively. Delta variants were detected from September - December of 2021 with occasional detections after December 2021, while Omicron variants were detected from January 2022-October 2023.

**Conclusion:**

Compared to Delta variants, Omicron variants were detected among children with medically attended ARI for a longer period and included more viral diversity. Continued monitoring of COVID-19 variants and their impact on pediatric illness is important.

**Disclosures:**

Rangaraj Selvarangan, BVSc, PhD, D(ABMM), FIDSA, FAAM, Abbott: Grant/Research Support|Abbott: Honoraria|BioMerieux: Grant/Research Support|Cepheid: Grant/Research Support|Diasorin: Grant/Research Support|GSK: Advisor/Consultant|Hologic: Grant/Research Support|Luminex: Grant/Research Support|Qiagen: Grant/Research Support

